# Envisioning a Global Health Partnership Movement

**DOI:** 10.1186/s12992-015-0138-4

**Published:** 2016-01-06

**Authors:** Andrew Jones

**Affiliations:** Tropical Health & Education Trust (THET), London, UK

“A universal truth: No health without a workforce” was the rallying cry of the flagship report commissioned by the Global Health Workforce Alliance Secretariat and the World Health Organization [1] and one which must be embraced if the aspiration for universal health coverage is ever to be realised [2]. One in seven people will never see a qualified health worker in their lives. The world will be short of 12.9 million health-care workers by 2035. The figures speak for themselves. It has never been clearer that there has to be a major global effort to recruit, educate and train health workers.

As the international development community prepare for the delivery of the next set of development goals, focus must include a meaningful revitalisation of the concept of partnership and a shift from short-term global interests to strengthening systems in low and middle-income countries. The Sustainable Development Goals call on new forms of partnership that speak to co-development rather than traditional models of international development - mutuality, co-learning and a recognition that we gain as much as we give by working through partnerships. It is time for donors and governments to look beyond monetary contributions to also consider what resources, expertise and technology that, if shared, could result in mutual benefit. In this sense, health partnerships offer a vision of the way in which learning and knowledge-exchange will take place in the future.

Among those engaged in global health, there is growing interest in long-term, formalised partnerships or links between health delivery and health education institutions such as hospitals, universities and professional associations. These partnerships are often developed by institutions with complementary objectives and are typically, but not exclusively, made up of health professionals from high-resource settings who volunteer their time and expertise to deliver health workforce strengthening or related projects. In many cases, partnerships are a two-way exchange of experience, skills and expertise from a high-income institution aiming to build the capacity of a low-income counterpart. However, multi-country and, increasingly, south-to-south partnership arrangements also exist. The themes, specialisms and methods of delivery of projects are broad, ranging from training and capacity building for in-service staff, providing practical skills, e-learning, online mentoring, continuing professional development, to facilitating research, professional standards and curriculum development to strengthen pre-service training.

We initiated this special series to explore these peer-to-peer collaborations and to start to build an accessible and shared knowledge base on their ability to offer an effective framework for sharing expertise internationally. It’s not just about development and capacity building; the series will underscore the additional benefits, challenges, and opportunities of taking multi-national partnership approaches. We anticipate that as the series evolves, it will become clearer where the gaps in our knowledge and evidence base exist and how we might address these.

We were very pleased to discover that our initial call for papers generated interest from all over the world and that a great diversity of health specialties and approaches are represented. This series is intended to be an on-going collection of evidence, reflections, and challenges to the health partnership approach. In this issue we begin with six articles and introduce some of the key questions for future exploration.

Over the past five years, the Health Partnership Scheme (HPS), a UK government-funded programme, has allowed for a variety of projects in different contexts and technical areas to be supported with appropriate levels of funding - the specifics of delivery are identified at the institutional level. Over 40,000 health workers have availed of training course places in over 29 countries in sub-Saharan Africa, Asia and the Middle East, through the voluntary engagement of more than 1,500 professionals from the UK’s National Health Service (NHS) and the involvement of more than 200 UK and overseas hospitals, universities and professional associations. HPS represents an important opportunity to gather evidence on a larger scale on what works well and what does not in a health partnership approach to capacity building. Suzanne Edwards et al. in seeking to develop a simple typology of international health partnerships, present initial analysis of 54 successful project proposals for health partnerships funded by HPS in 2012–2013. The variability of partnerships contributes to the challenge of understanding their effectiveness and a typology of partnerships could aid evaluation [3].

Glorieuse Uwizeye et al. assess the twinning model in the Rwandan Human Resources for Health Program, exploring the United States teaching institutions and Rwandan Faculty goal setting, satisfaction and perceptions of the effectiveness of skill transfer [4].

Increasing levels of interest in a capacity building approach have led to questions about the mechanisms, efficiency and effectiveness of health partnerships, prompting a small but rising stream of published evaluations and research papers. A rapid evidence review on the effectiveness of institutional health partnerships is presented by Ema Kelly et al. [5].

Ben Hague et al. examine the individual and organisational benefits of a partnership between a National Health Service (NHS) mental health Trust in the United Kingdom and a mental health referral hospital in Northern Uganda [6]. Lawrence C. Loh et al. review short term global health experiences and local partnership models and propose a framework to categorise different models of local partner engagement [7]. Barbara Anne Jack et al. reports on an initiative where palliative care staff, both clinical and academic, volunteered to help to develop, support and deliver a degree in palliative care in sub-Saharan Africa. The objective of the study was to explore the personal impact on the health care professionals of being part of this initiative [8].

Evidence about the effectiveness of health partnerships is largely qualitative, particularly when it comes to evidencing more complex aspects of training and capacity building such as longer-term changes in health worker performance. Nevertheless, some interventions have been able to demonstrate the effectiveness of their work, not least in terms of tangible outputs and indications of longer-term change, but also evidence of the ways in which their work offers a value-for-money approach. For example, two case studies commissioned by THET, examine the ways in which health partnerships provide a value for money approach to health workforce development [9].

There is need for further research worldwide on what partnerships can achieve, examples of failure and on what makes an effective partnership. The breadth of questions should engage clinicians, social scientists, health educationalists, global health specialists, economists and others, but while many partnerships publish on their own experiences and clinical findings, there has been little effort to align research activities that examine health partnerships as a mechanism for delivery. THET’s grounding in practice and our connections with both practitioners and researchers put us in a unique position as a hub for research into health partnerships, and we welcome contact from researchers or practitioners exploring these and further issues.

## Key questions for future research

- How can we talk about the collective impact of the health partnership model or approach, given the diversity of institutions, relationships, health system contexts and interventions?
- Under what circumstances do health partnerships offer an efficient and effective way to deliver health workforce strengthening interventions?
- How do health partnerships compare with other models for delivering health workforce strengthening interventions?
- How can health partnerships measure the value of health workforce strengthening interventions?
- How can we measure the value of a health partnership approach beyond supporting the delivery of health workforce strengthening interventions, with particular reference to social capital?
